# Respiratory Muscle Training Combinations in Amateur Runners: A Randomized Trial of Pulmonary Function, Respiratory Muscle Strength, and Exercise Capacity

**DOI:** 10.3390/bioengineering13010011

**Published:** 2025-12-23

**Authors:** Eunho Lee, Jinseop Kim

**Affiliations:** Department of Physical Therapy, Sunmoon University, Asan-si 31460, Republic of Korea; red66y@naver.com

**Keywords:** pulmonary ventilation, exercise tolerance, resistance training, cardiopulmonary exercise testing, running, respiratory muscles

## Abstract

Background: Amateur runners may benefit from combining respiratory muscle training (RMT) with resistance or aerobic modalities, but direct comparisons are scarce. This study compared different RMT-based combinations on pulmonary function, respiratory muscle strength, and whole-body exercise capacity. Methods: In this randomized four-arm trial, 48 amateur runners were allocated equally to stand-alone RMT, RMT plus upper-limb resistance (RMT + ULRT), RMT plus lower-limb resistance (RMT + LLRT), or RMT plus aerobic exercise (RMT + AET). All groups completed supervised sessions three times per week for six weeks. Pulmonary function (forced vital capacity [FVC], forced expiratory volume in one second [FEV_1_], FEV_1_/FVC), respiratory muscle strength (maximal inspiratory and expiratory pressures, MIP and MEP), and cardiopulmonary exercise test indices (peak oxygen uptake [VO_2_peak], VE/VCO_2_ slope) were assessed before and after training using standardized spirometry, mouth-pressure measurements, and treadmill cardiopulmonary exercise testing (CPET). Pre–post changes within groups and the overall between-group differences were evaluated using standard parametric methods. Results: All four interventions were associated with improvements in at least one respiratory or cardiopulmonary domain. FVC and FEV_1_ tended to improve more in the resistance-combination groups, whereas the FEV_1_/FVC ratio increased with RMT alone and when combined with resistance. MIP increased in the RMT, RMT + ULRT, and RMT + LLRT groups, and MEP increased across all groups. VO_2_peak rose in every group, while the VE/VCO_2_ slope improved only when RMT was combined with upper- or lower-limb resistance or aerobic exercise. Between-group differences in change scores were not statistically significant and did not clearly favor any single regimen. Conclusions: In amateur runners, six weeks of RMT-based programs are feasible and associated with domain-specific improvements in lung function, respiratory muscle strength, and exercise capacity. Because between-group differences in change scores were not statistically significant and the sample size was modest, these findings should be considered exploratory and may inform hypothesis generation regarding the use of different RMT combinations in future, larger trials.

## 1. Introduction

Running performance and injury risk in amateur runners are influenced by training load, intensity, and how training volume progresses over time. Compared with world-class distance runners, recreational runners often follow less structured periodization; their outcomes are closely related to total weekly volume, longest-run duration, and week-to-week load changes [1,2,3]. In practice, training commonly emphasizes lower-limb strength and aerobic capacity, whereas respiratory muscle function and ventilatory efficiency receive less attention, despite the importance of monitoring training dose in this population [4].

Respiratory muscle fatigue can act as an independent limiter of high-intensity and sustained exercise. A substantial body of evidence shows that respiratory muscle training (RMT), including inspiratory muscle training (IMT), improves inspiratory and expiratory strength (MIP/MEP), pulmonary function, and functional capacity across clinical populations [5,6,7,8]. Beyond clinical settings, RMT has been linked to upward shifts in lactate threshold and improved high-intensity performance in healthy participants and athletes [9,10,11,12,13], and a recent randomized trial in amateur runners reported IMT-related attenuation of lactate accumulation alongside performance gains [14]. When RMT is combined with other modalities, additional pathways may be targeted: aerobic exercise and combined rehabilitation frequently improve ventilatory efficiency (lower VE/VCO_2_ slope) and exercise tolerance [15,16,17,18], whereas upper- or lower-limb resistance training can enhance thoracic expansibility, trunk/pelvic stabilization, and accessory-muscle recruitment, thereby supporting more economical breathing mechanics [19,20,21,22,23,24]. At the same time, protocol heterogeneity and short intervention windows often yield small or inconsistent changes in spirometric indices, and practical constraints—such as transient post-session decrements in respiratory strength after high-volume resistance work or device-related psychophysiological responses—counsel careful periodization and monitoring [25,26]. Related clinical literature (e.g., pulmonary hypertension, obstructive sleep apnea) strengthens the plausibility of efficiency-focused mechanisms but also illustrates variability across populations and protocols [27,28].

Head-to-head comparisons of respiratory muscle training (RMT) alone versus RMT combined with upper-limb resistance training (ULRT), lower-limb resistance training (LLRT), or aerobic exercise training (AET) under a unified protocol are scarce in healthy amateur runners. Addressing this gap, the present randomized four-arm trial compares these strategies to describe their effects on pulmonary function (forced vital capacity [FVC], forced expiratory volume in one second [FEV_1_], FEV_1_/FVC ratio), respiratory muscle strength (maximal inspiratory and expiratory pressures, MIP and MEP), whole-body aerobic capacity (peak oxygen uptake [VO_2_peak]), and ventilatory efficiency (VE/VCO_2_ slope) in amateur runners. Rather than establishing superiority of any single regimen, this study aims to characterize the within-group response patterns associated with different RMT-based combinations and to generate hypotheses for future, larger trials.

## 2. Materials and Methods

**Figure 1:**
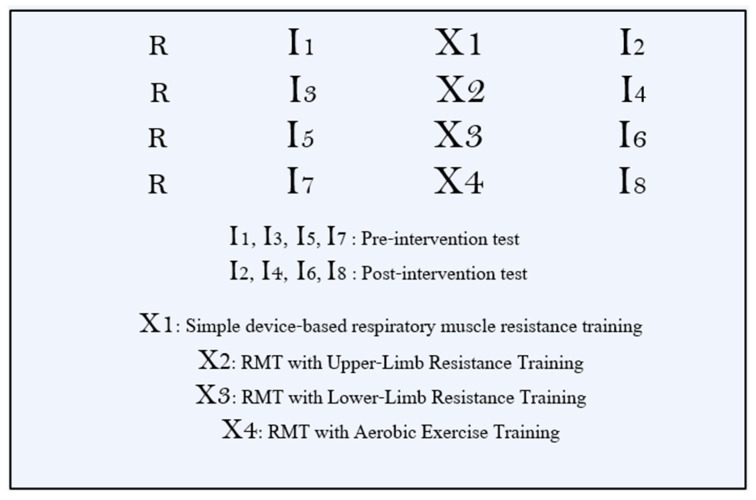
Study design.

This study used a randomized, four-arm, parallel, pre–post design to compare (i) stand-alone respiratory muscle training (RMT), (ii) RMT plus upper-limb resistance training (RMT + ULRT), (iii) RMT plus lower-limb resistance training (RMT + LLRT), and (iv) RMT plus aerobic exercise training (RMT + AET). All outcomes were assessed during two dedicated laboratory visits: a baseline (pre-intervention) assessment before the start of the 6-week program and a post-intervention assessment after completion of the 6-week program, using identical procedures at both time points. No additional daily or weekly outcome measurements were collected during the intervention; the thrice-weekly sessions were reserved for training. Participants were not prescribed a standardized running program during the 6-week period. Therefore, overall running training load (e.g., weekly volume and intensity) and any concurrent strength training outside the supervised sessions were not formally monitored or controlled. After baseline assessments, participants were randomly assigned in a 1:1:1:1 ratio to the RMT, RMT + ULRT, RMT + LLRT, or RMT + AET group using a simple drawing-lots procedure without stratification. Randomization was performed by an investigator who was not involved in outcome assessments, and outcome assessors were kept unaware of group allocation to maintain a single-blind design. In Figure 1, R denotes randomization. The protocol received approval from the Institutional Review Board of Sun Moon University (SM-202509-017-2), and all participants provided written informed consent prior to enrollment.

Eligible participants were adults aged 20–45 years who met an operational definition of “amateur runner”: they had run at least twice per week for ≥6 months and averaged 15–80 km per week over the preceding 3 months, with non-elite competitive status. Exclusion criteria comprised very low recent running exposure (<5 km/week over the past 3 months or <10 km total in the past year), medical contraindications to exercise involving cardiovascular, respiratory, neurological, or musculoskeletal systems, and any investigator-determined factors (e.g., medication or alcohol use) that could render participation inappropriate.

**Figure 2:**
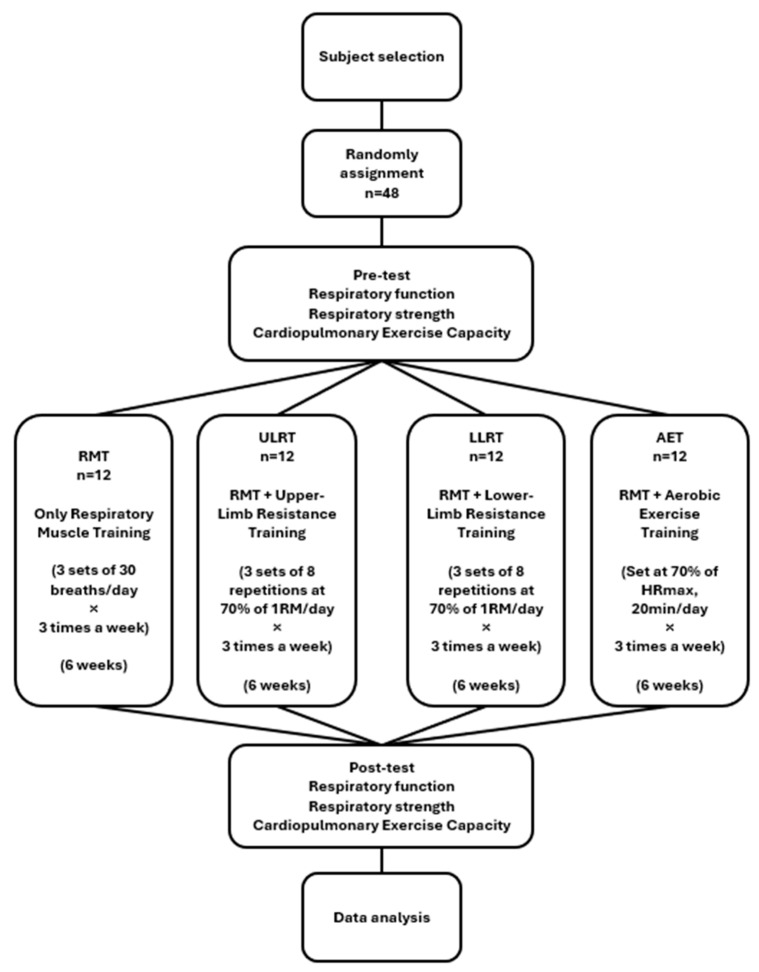
Flow diagram of the study procedure.

An a priori power analysis (G*Power 3.1.9.7) for a one-way ANOVA on change scores (Δ = post − pre; four groups) assumed an effect size of f = 0.50, α = 0.05 (two-tailed), and power (1 − β) = 0.80, yielding a required sample of *n* = 12 per group (total N = 48). Given the modest per-group sample size and limited head-to-head evidence, this trial was designed as exploratory (pilot-scale) and hypothesis-generating rather than confirmatory for establishing between-group superiority. The assumed effect size (f = 0.50) was selected to represent a moderate-to-large between-group difference, informed by previous trials reporting clinically relevant improvements in spirometric indices, respiratory muscle strength, and VO_2_peak following respiratory muscle training or combined aerobic–resistance interventions in adults [5,6,7,8,9,10,11,12,13,14,29]. Eligibility was prescreened; after consent, baseline characteristics (sex, date of birth, height, weight), health status (recent illness, injuries, medications), and self-reported running training habits (e.g., sessions per week and weekly running distance) were recorded. Participants were then randomly assigned by drawing lots to one of the four groups (*n* = 12 each), and assessors remained unaware of allocation to the extent possible. The flow diagram of the study procedure is presented in Figure 2.

**Figure 3:**
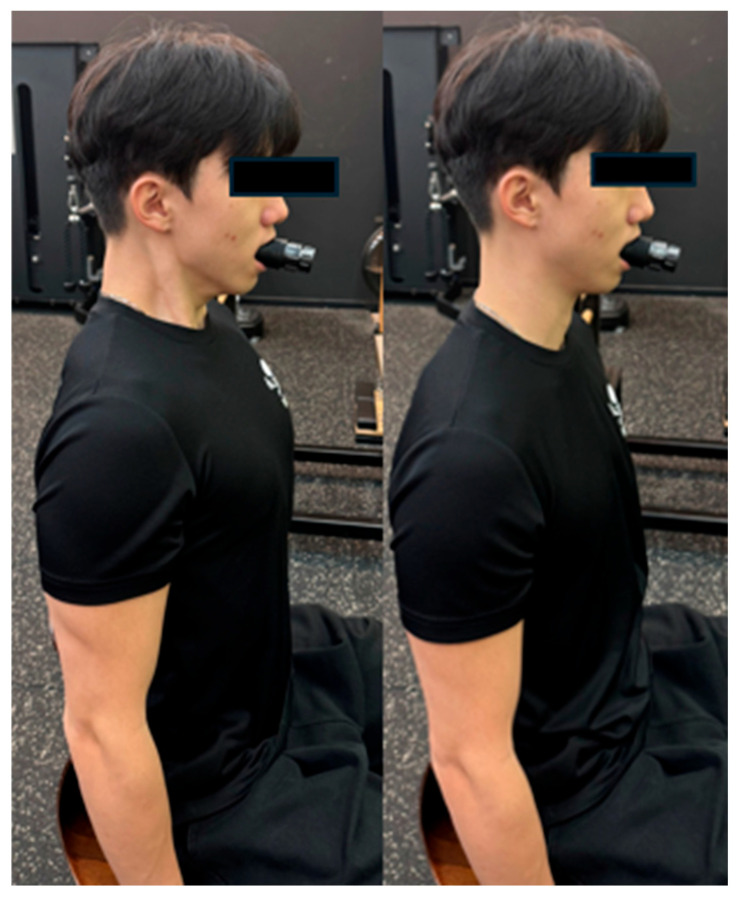
Respiratory Muscle Training.

All participants completed a six-week program consisting of three supervised sessions per week. Respiratory muscle training (RMT) formed the common core across arms and was delivered with a pressure-threshold inspiratory muscle training device (Threshold, Yiwu Shimai Trade Co., Ltd, Yiwu, China) while seated with back support. Each session comprised three sets of 30 breaths with 30 s inter-set rest. The initial inspiratory load was set at approximately 30% of each participant’s baseline maximal inspiratory pressure (MIP). The inspiratory load was then progressed by about 5–10% of MIP between sessions when the participant could comfortably complete all three sets of 30 breaths using a stable diaphragmatic breathing pattern (full expiration → deep inspiration → full expiration) at a target rating of perceived exertion (RPE) of 13–15, without dizziness, headache, chest discomfort, or disproportionate dyspnea. Sessions were paused or terminated if dizziness, headache, chest discomfort, or disproportionate dyspnea occurred. Standardized warm-up and cool-down were applied in all arms, and adherence was monitored with attendance logs and home record sheets. Adherence was operationalized as attendance at the supervised sessions (18 planned sessions over six weeks) and was tracked using session attendance logs. Because all training was supervised, completion of the prescribed sets/repetitions/breaths and tolerance to the session were checked in real time by study staff, and any deviations (e.g., pausing/termination due to symptoms) were noted. For the RMT component, protocol fidelity was supported by standardized progression criteria (load increased by ~5–10% of baseline MIP when participants completed all sets with stable diaphragmatic breathing at target RPE 13–15 and without disproportionate symptoms). For the aerobic arm, heart rate was continuously monitored and RPE was recorded to verify internal load at the prescribed intensity. The respiratory muscle training procedure is illustrated in Figure 3.

**Figure 4:**
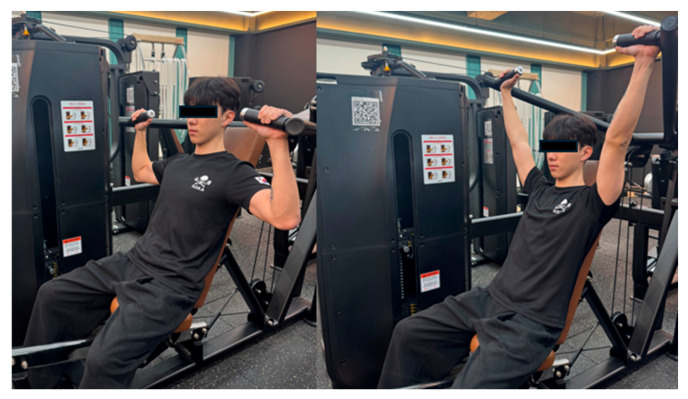
Shoulder press.

In the RMT + ULRT arm, the RMT session was followed—after a 3 min seated recovery—by upper-limb resistance exercise emphasizing scapulothoracic control (shoulder press; LS-701; LEXCO, Daegu, Republic of Korea). Loads were prescribed at 70% of one-repetition maximum (1RM), performed for 3 sets of 8 repetitions with 60 s inter-set rest. One-repetition maximum for the shoulder press was determined at baseline using a supervised incremental loading test and defined as the highest load that could be lifted once with full range of motion and proper technique; the 70% 1RM load was then used to prescribe training intensity across the 6-week intervention. Repetitions were executed through the full range of motion without momentum, with coaching cues to maintain scapulothoracic rhythm and avoid excessive rib-cage elevation. The shoulder press exercise setup is illustrated in Figure 4.

**Figure 5:**
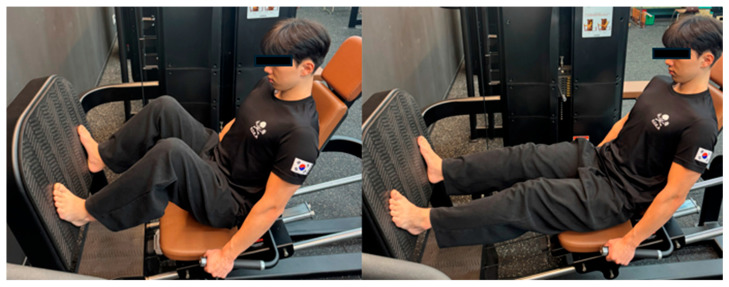
Leg press.

In the RMT + LLRT arm, participants completed the same RMT protocol and then—after a 3 min recovery—performed lower-limb resistance exercise on a legpress (LS-117; LEXCO, Daegu, Republic of Korea) at 70% 1RM, 3 × 8 with 60 s inter-set rest. For the legpress exercise, 1RM was likewise assessed at baseline with a progressive loading procedure under supervision and training loads were set at 70% of this baseline 1RM value for the duration of the program. Technique instruction prioritized alignment, lumbopelvic stability, and coordinated breathing to support intra-abdominal pressure and lower-extremity–core coupling (Figure 5).

**Figure 6:**
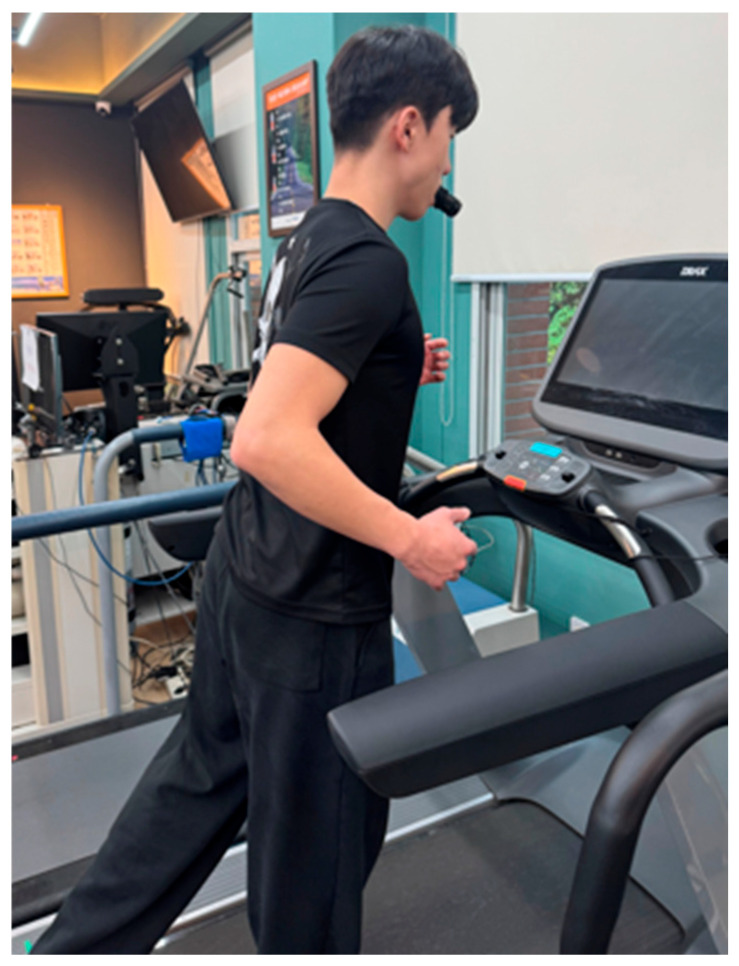
Treadmill training.

In the RMT + AET arm, RMT was followed—after a 3 min recovery—by treadmill(NR20; DRAX Inc., Anyang-si, Gyeonggi-do, Republic of Korea)-based aerobic exercise at 70% of age-predicted maximal heart rate for 20 min per session. Each bout included a 3–5 min warm-up to the target intensity, steady-state maintenance at the prescribed workload, and a 3–5 min cool-down. Heart rate was continuously monitored, and ratings of perceived exertion (RPE 13–15) were recorded in parallel to verify internal load (Figure 6).

**Figure 7:**
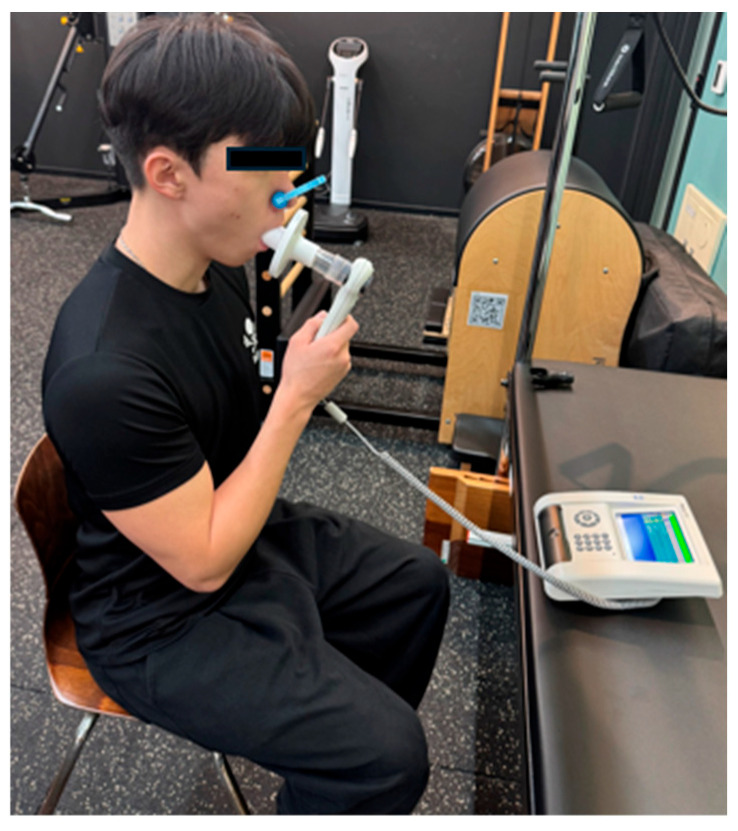
Spirometry.

Respiratory function and respiratory muscle strength were assessed using standard spirometry (PONY FX, COSMED, Albano Laziale, Italy) and mouth-pressure maneuvers. Forced vital capacity (FVC, L) was defined as the total volume of air forcibly exhaled after a full inspiration, providing an index of overall ventilatory capacity. Forced expiratory volume in one second (FEV_1_, L) was defined as the volume exhaled during the first second of the FVC maneuver, reflecting expiratory flow and large-airway function. The FEV_1_/FVC ratio expressed the proportion of the vital capacity exhaled in the first second and was used to screen for obstructive ventilatory patterns. Maximal inspiratory pressure (MIP, mmH_2_O) and maximal expiratory pressure (MEP, mmH_2_O) were measured at the mouth as the peak inspiratory pressure from near residual volume and the peak expiratory pressure from near total lung capacity, respectively, and were used as global indices of inspiratory and expiratory respiratory muscle strength. For spirometry and respiratory pressures, three technically acceptable trials were obtained for each outcome, and the highest value was retained for analysis (Figure 7).

**Figure 8:**
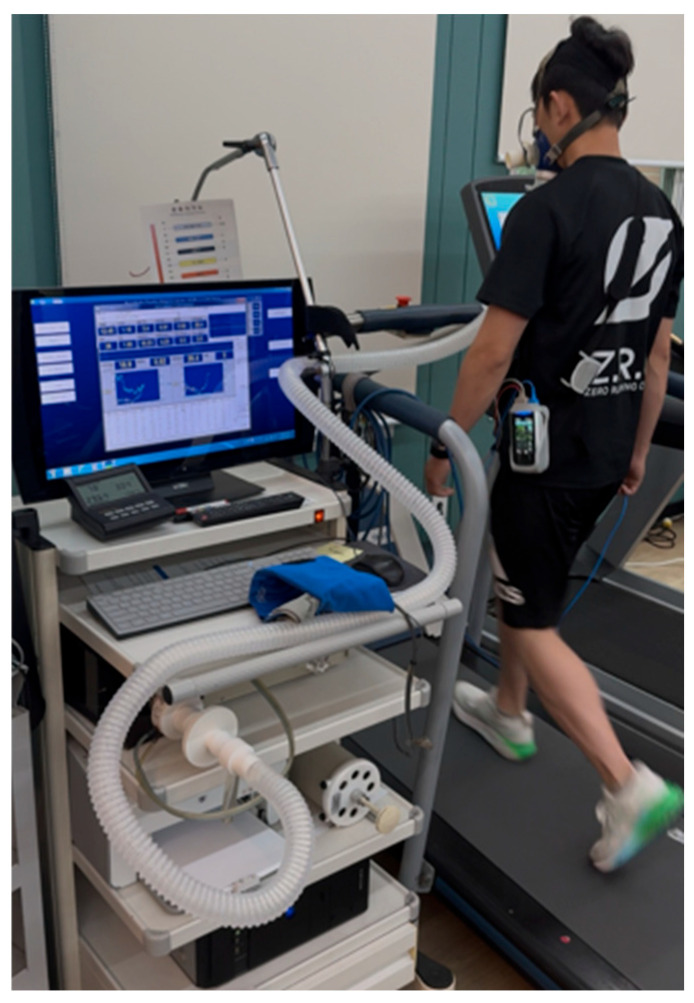
Cardiopulmonary exercise testing.

Whole-body aerobic capacity and ventilatory efficiency were measured by cardiopulmonary exercise testing (CPET). From breath-by-breath gas exchange, peak oxygen uptake (VO_2_peak) and the ventilatory equivalent for carbon dioxide slope (VE/VCO_2_ slope) were derived. Before each test, the metabolic cart and flowmeter were calibrated per manufacturer guidelines, and a size-appropriate facemask was fitted to ensure an airtight seal. Cardiopulmonary exercise testing was performed on a motorized treadmill using the Modified Bruce Protocol, which begins with two low-intensity 3 min stages at 1.7 mph with 0% and 5% grade, respectively, followed by the standard incremental Bruce stages until volitional exhaustion [30]. Ratings of perceived exertion (RPE) were monitored continuously, and all test procedures and termination criteria were explained in advance. Whole-body aerobic capacity was expressed as peak oxygen uptake (VO_2_peak, mL·kg^−1^·min^−1^), defined as the highest 15–30 s time-averaged oxygen uptake value attained during the treadmill cardiopulmonary exercise test and normalized to body mass. VO_2_peak was selected as the primary index of aerobic performance in amateur runners. VO_2_peak and VE/VCO_2_ slope were selected a priori as complementary CPET outcomes to capture whole-body aerobic capacity and ventilatory efficiency, both of which are physiologically relevant to endurance exercise in runners. These indices were expected to be responsive over a six-week period because improvements in respiratory muscle strength with RMT may reduce the relative ventilatory burden and influence ventilatory efficiency during exercise, potentially affecting CPET-derived responses even when spirometric changes are modest. This rationale aligns with the mechanistic framework described in the Introduction regarding RMT-related adaptations in respiratory muscle performance and exercise tolerance. Ventilatory efficiency was quantified using the VE/VCO_2_ slope, calculated as the slope of the linear relationship between minute ventilation (VE) and carbon dioxide output (VCO_2_) from breath-by-breath data from rest to peak exercise. Because VE/VCO_2_ slope is less dependent on achieving a true maximal effort than VO_2_peak, it provides complementary information to VO_2_peak in characterizing exercise responses (Figure 8).

In accordance with the study hypotheses examining the effects of respiratory muscle training (RMT) and combined exercise modalities on pulmonary function, respiratory muscle strength, and whole-body exercise capacity, the present study was conducted as follows (Table 1).

Data were analyzed using IBM SPSS Statistics for Windows (version 26.0; IBM Corp, Armonk, NY, USA). All continuous variables are presented as mean ± standard deviation. Statistical significance was set at two-tailed *p* < 0.05. No formal correction for multiple comparisons was applied. Therefore, *p*-values should be interpreted as nominal and exploratory, particularly given the number of outcomes assessed. Prior to analysis, assumptions of normality (Shapiro–Wilk) and homogeneity of variance (Levene’s test) were assessed. Within-group changes were evaluated using paired-samples *t*-tests comparing pre- and post-intervention values. Between-group differences were examined with one-way ANOVA on change scores (Δ = post − pre) across the four groups. Analyses included participants who completed both baseline and post-intervention assessments; cases with missing data were excluded.

## 3. Results

### 3.1. General Characteristics of Subjects

A total of 48 participants (12 per group) completed both pre- and post-intervention assessments, which met the a priori sample size target (power 1 − β = 0.80 at α = 0.05 for an assumed effect size f = 0.50). The general characteristics of the participants are presented in Table 2. Of the sample, 31 participants (64.6%) were men. The mean age was 32.98 ± 4 years; mean height, 170.44 ± 9.32 cm; mean body mass, 69.53 ± 13 kg; and body mass index, 23.77 ± 2.87 kg/m^2^. Participants reported 3.35 ± 1.18 running sessions per week and a weekly running distance of 28.15 ± 18.33 km. Twelve participants (25%) were current smokers. Baseline characteristics by group are summarized in Table 2. There were no statistically significant differences among the four groups in age, sex distribution, body mass index, smoking status, or running training habits (weekly running frequency, duration, and distance).

Baseline comparability of key outcome variables is shown by the pre-test between-group *p*-values in Table 3, Table 4, Table 5, Table 6, Table 7, Table 8 and Table 9. At baseline, groups were comparable for FVC (*p* = 0.48), FEV_1_ (*p* = 0.70), FEV_1_/FVC (*p* = 0.23), MIP (*p* = 0.12), MEP (*p* = 0.20), and VE/VCO_2_ slope (*p* = 0.74). However, VO_2_peak differed significantly between groups at baseline (*p* = 0.03); therefore, VO_2_peak between-group interpretations are presented as descriptive and exploratory.

### 3.2. Respiratory Function

#### 3.2.1. Forced Vital Capacity (FVC)

In this study, between-group comparisons for FVC showed no statistically significant differences in baseline means among the four groups (*p* = 0.48). Post-intervention between-group differences in means were likewise nonsignificant (*p* = 0.63). Within-group pre–post analyses indicated significant increases in FVC for RMT + ULRT (0.31 ± 0.28; *p* < 0.001) and RMT + LLRT (0.30 ± 0.34; *p* = 0.01). By contrast, RMT alone (0.21 ± 0.47; *p* = 0.15) and RMT + AET (0.05 ± 0.31; *p* = 0.57) showed no significant change. The between-group comparison of change scores (ΔFVC) was not significant (*p* = 0.29), although within-group improvements were confirmed in the two resistance-training combination groups (Table 3).

#### 3.2.2. Forced Expiratory Volume in One Second (FEV_1_)

In this study, between-group comparisons for FEV_1_ revealed no statistically significant differences in baseline means among the four groups (pre-test *p* = 0.70), and post-intervention between-group mean differences were likewise nonsignificant (post-test *p* = 0.58). Within-group pre–post analyses showed a significant increase only in the RMT + LLRT group (0.19 ± 0.14; *p* < 0.001), whereas RMT (0.08 ± 0.30; *p* = 0.36), RMT + ULRT (0.07 ± 0.14; *p* = 0.10), and RMT + AET (0.11 ± 0.24; *p* = 0.13) exhibited no significant change. The between-group comparison of change scores (ΔFEV_1_) was also nonsignificant (*p* = 0.51), indicating that a within-group improvement in FEV_1_ was confirmed only when lower-limb resistance training was combined with RMT (Table 4).

#### 3.2.3. The Ratio of Forced Expiratory Volume for 1 s to Forced Vital Capacity (FEV_1_/FVC)

In the analysis of the FEV_1_/FVC ratio, between-group comparisons were nonsignificant at both baseline (pre-test *p* = 0.23) and post-test (*p* = 0.29). However, within-group pre–post changes were significant in the RMT group (+2.17 ± 2.21%; *p* = 0.006), the RMT + ULRT group (+1.58 ± 1.68%; *p* = 0.007), and the RMT + LLRT group (+1.25 ± 1.42%; *p* = 0.011). By contrast, the RMT + AET group showed an increase of +2.42 ± 4.78% that did not reach statistical significance (*p* = 0.107). The between-group comparison of change scores (ΔFEV_1_/FVC) was also nonsignificant (change *p* = 0.74). Thus, the ratio improved significantly within three interventions (RMT, RMT + ULRT, RMT + LLRT) (Table 5).

### 3.3. Respiratory Strength

#### 3.3.1. Maximal Inspiratory Pressure (MIP)

In the MIP analysis, baseline between-group means did not differ (pre-test *p* = 0.12), and post-test between-group mean differences were likewise nonsignificant (post-test *p* = 0.41). Within-group pre–post changes were significant in the RMT group (increase of 18.92 ± 11.52; *p* < 0.001), the RMT + ULRT group (16.42 ± 22.01; *p* = 0.03), and the RMT + LLRT group (11.75 ± 10.01; *p* < 0.001). By contrast, the RMT + AET group showed no significant change (2.92 ± 14.37; *p* = 0.50). The between-group comparison of change scores (ΔMIP) approached but did not reach significance (change *p* = 0.07). Overall, significant increases in MIP were observed in all RMT-based groups except the aerobic-combination group (Table 6).

#### 3.3.2. Maximal Expiratory Pressure (MEP)

In the MEP analysis, baseline between-group means did not differ (pre-test *p* = 0.20), and post-test between-group mean differences were likewise nonsignificant (post-test *p* = 0.47). Within-group pre–post changes were significant in all four groups: RMT (increase of 10.92 ± 17.97; *p* < 0.001), RMT + ULRT (16.92 ± 14.21; *p* < 0.001), RMT + LLRT (15.08 ± 15.32; *p* < 0.001), and RMT + AET (2.17 ± 20.16; *p* < 0.001). However, the between-group comparison of change scores (ΔMEP) was not significant (change *p* = 0.17). Overall, all groups exhibited improvements in expiratory muscle strength (Table 7).

### 3.4. Whole-Body Exercise Capacity

#### 3.4.1. Peak Oxygen Uptake (VO_2_peak)

In the VO_2_peak analysis, baseline between-group means differed significantly (pre-test *p* = 0.03; RMT 36.17 ± 6.58, RMT + ULRT 37.25 ± 6.84, RMT + LLRT 43.48 ± 6.06, RMT + AET 40.26 ± 6.08), and post-test between-group differences remained significant (post-test *p* = 0.01; RMT 40.76 ± 6.38, RMT + ULRT 40.86 ± 6.27, RMT + LLRT 48.58 ± 7.29, RMT + AET 46.04 ± 5.99). Within-group pre–post changes were significant in all four groups: RMT +4.59 ± 1.57 (*p* < 0.001), RMT + ULRT +3.61 ± 1.53 (*p* < 0.001), RMT + LLRT +5.10 ± 5.50 (*p* < 0.01), and RMT + AET +5.78 ± 3.49 (*p* < 0.001). However, the between-group comparison of change scores (ΔVO_2_peak) was not significant (change *p* = 0.47), indicating that all four prescriptions improved VO_2_peak without establishing statistical superiority of any single group (Table 8). Moreover, because no baseline-adjusted analysis (e.g., ANCOVA) was applied despite the significant pre-test imbalance, all VO_2_peak comparisons between groups should be interpreted as descriptive and exploratory rather than as definitive estimates of differential intervention efficacy.

#### 3.4.2. Minute Ventilation–Carbon Dioxide Slope (VE/VCO_2_ Slope)

In the analysis of the VE/VCO_2_ slope, between-group means did not differ at baseline (pre-test *p* = 0.74) and remained nonsignificant at post-test (*p* = 0.49). Within-group pre–post changes showed significant decreases (improvements) in all combined groups except RMT alone: RMT + ULRT −1.94 ± 1.27 (*p* < 0.001), RMT + LLRT −2.68 ± 2.42 (*p* < 0.001), and RMT + AET −1.42 ± 1.49 (*p* = 0.01). By contrast, RMT alone showed no significant change (−0.48 ± 3.45; *p* = 0.64). The between-group comparison of change scores (ΔVE/VCO_2_ slope) was also nonsignificant (change *p* = 0.14), indicating that while ventilatory efficiency improved across combined interventions, no statistical superiority of any single group was established (Table 9).

Taken together, the pre–post analyses across all four groups showed that the 6-week interventions produced meaningful improvements in at least one key domain (lung function, respiratory muscle strength, or exercise capacity) in all groups, although the pattern and magnitude of change differed between interventions (Table 3, Table 4, Table 5, Table 6, Table 7, Table 8 and Table 9). Lung function indices (FVC and FEV_1_) showed modest pre–post improvements, with statistically significant gains observed mainly in the combined resistance groups (RMT + ULRT and RMT + LLRT for FVC and RMT + LLRT for FEV_1_), whereas the FEV_1_/FVC ratio remained within the normal range and showed only small, clinically negligible increases without between-group differences, indicating no development of obstructive ventilatory patterns. Respiratory muscle strength (MIP and MEP) improved significantly in the RMT, RMT + ULRT and RMT + LLRT groups for MIP and in all four groups for MEP, suggesting that both isolated RMT and RMT combined with resistance or aerobic training can enhance respiratory muscle performance. For exercise capacity, VO_2_peak increased significantly from pre- to post-intervention in all four groups, with descriptively larger gains in the RMT + LLRT and RMT + AET groups compared with RMT alone, while changes in VE/VCO_2_ slope showed significant reductions (improvements) in ventilatory efficiency in the three combined groups (RMT + ULRT, RMT + LLRT and RMT + AET) but not in the RMT-only group. However, between-group comparisons of change scores did not reveal statistically significant differences for any outcome, although numerically larger improvements in VO_2_peak and VE/VCO_2_ slope tended to favor the RMT + LLRT and RMT + AET groups, indicating that the different combinations of RMT, resistance training, and aerobic training exert partially overlapping but not identical benefits on respiratory and cardiopulmonary function.

From a practical perspective for amateur runners, the magnitude of change suggests potentially meaningful physiological adaptation over six weeks. VO_2_peak increased across all interventions by approximately +3.61 to +5.78 mL·kg^−1^·min^−1^, indicating improved aerobic capacity within each training approach. Ventilatory efficiency also improved in the combined interventions, as reflected by reductions in VE/VCO_2_ slope (approximately −1.42 to −2.68), while the RMT-only group showed no significant change. However, because between-group Δ comparisons were not significant and baseline-adjusted models were not applied (notably for VO_2_peak, which showed baseline imbalance), these patterns should be interpreted as descriptive and exploratory rather than evidence of superiority of any modality. In addition, spirometric ratio changes (FEV_1_/FVC) were small and clinically negligible, and running-specific performance outcomes were not measured; therefore, practical implications are limited to physiological signals rather than confirmed improvements in running performance.

## 4. Discussion

This randomized, four-arm trial evaluated six weeks of a standardized respiratory muscle training (RMT) program performed alone or combined with upper-limb resistance (RMT + ULRT), lower-limb resistance (RMT + LLRT), or aerobic exercise (RMT + AET) in amateur runners. Across all groups, we observed within-group pre–post improvements in at least one domain: pulmonary function (FVC, FEV_1_, FEV_1_/FVC), respiratory muscle strength (MIP, MEP), or CPET-derived indices (VO_2_peak, VE/VCO_2_ slope). The pattern of change differed by group, but between-group comparisons of change scores were not statistically significant and baseline VO_2_peak differed among arms. Therefore, these findings should be interpreted as exploratory and hypothesis-generating rather than as evidence of modality-specific superiority. Within this framework, our results fit into the broader context in which world-class practice emphasizes periodized integration of aerobic capacity, strength, and technique [1], while recreational runners’ outcomes are closely linked to weekly volume, longest endurance run, and load progression [2,3,4,31]. Accordingly, any apparent emphasis of specific domains in particular combinations (for example, slightly larger within-group changes in spirometric indices with resistance pairings or in VO_2_peak and VE/VCO_2_ with the aerobic pairing) should be viewed strictly as descriptive trends and not as evidence that one combination is more effective than the others. Because running-specific performance outcomes were not measured in this trial, the observed changes should be interpreted as short-term physiological adaptations rather than definitive improvements in running performance.

Mechanistically, the pattern of FVC and FEV_1_ responses in the resistance-combination groups is compatible with improved chest-wall mechanics and thoracoabdominal coordination. Upper-limb resistance work can enhance scapulothoracic stabilization and accessory inspiratory muscle recruitment, potentially supporting greater end-inspiratory expansion during forced maneuvers (as reflected in FVC gains with RMT + ULRT) [19,20,21,32]. Lower-limb resistance exercise may improve lumbopelvic fixation and intra-abdominal pressure, favoring more efficient rib-cage expansion and expiratory flow (FVC and FEV_1_ changes with RMT + LLRT) [22,23,24,33]. Prior studies have reported that resistance or concurrent training can favorably affect lung function and mechanics, particularly in overweight/obese or older adults [23,24,29,34,35]. In the present trial, these mechanistic interpretations remain tentative; given the absence of significant between-group Δ differences and the use of change-score ANOVA rather than baseline-adjusted models, they should be regarded as hypothesis-generating rather than as proof that any resistance-based combination is superior to the others or to RMT alone.

The respiratory strength results align with the established effects of RMT and inspiratory muscle training in healthy and athletic populations. Pressure-threshold loading elicits rapid neural and strength adaptations in the inspiratory muscles, which is consistent with the MIP gains observed in our RMT-based groups [5,6,7,8]. In non-clinical samples, including healthy individuals, team-sport athletes, swimmers, and strength athletes, RMT has been shown to increase respiratory muscle strength and to influence physiological determinants related to exercise tolerance (e.g., lactate responses) in some athletic contexts [9,10,11,12,13,29,36], and a randomized trial in amateur runners reported improvements in respiratory strength and exercise tolerance [14]; however, the present study did not assess direct running performance outcomes, and therefore no performance enhancement can be concluded. Within this context, our findings extend previous work by demonstrating that, across four RMT-based programs, amateur runners can achieve meaningful within-group improvements in MIP and MEP over six weeks, even though statistically significant differences between specific combinations were not detected. These effects are also broadly consistent with meta-analytic evidence for RMT in chronic cardiopulmonary and neurological conditions [15,16,17,18,27,28,37,38,39,40,41,42,43,44,45,46,47,48,49,50,51,52,53,54,55,56], but the present trial specifically addresses healthy amateur runners.

Cardiopulmonary responses showed consistent enhancement of aerobic capacity, with more selective changes in ventilatory efficiency. Taken together with the significant baseline differences in VO_2_peak and the absence of baseline-adjusted modeling, these cardiopulmonary findings—particularly for VO_2_peak—should be viewed as internally limited and exploratory, rather than as providing robust evidence of differential efficacy between the four interventions. VO_2_peak increased significantly in all four arms, suggesting that RMT alone and RMT combined with resistance or aerobic stimuli can improve whole-body exercise capacity in amateur runners, potentially by attenuating the respiratory muscle metaboreflex and improving perceived effort [9,10,14]. By contrast, the VE/VCO_2_ slope decreased only when RMT was combined with upper-limb resistance, lower-limb resistance, or aerobic training, whereas RMT alone showed no significant change. Similar patterns, with preferential improvements in ventilatory efficiency under combined or aerobic training, have been reported in cardiopulmonary rehabilitation settings where aerobic exercise and RMT are integrated [15,16,17,18,27,37,51,57,58,59,60]. Related evidence also exists in other clinical contexts where respiratory-focused exercise is paired with limb training [61,62,63,64]. However, our trial did not detect statistically significant between-group Δ differences in VO_2_peak or VE/VCO_2_ slope, and VO_2_peak was imbalanced at baseline. Consequently, any apparent modality-specific emphases—such as greater efficiency gains with combined arms—should be viewed as preliminary trends requiring confirmation in larger, methodologically optimized trials rather than as firm evidence for choosing one specific combination as inherently superior in runners.

Our findings also align with work suggesting that concurrent or combined training strategies can be tailored to emphasize different physiological targets. Studies in physically active adults, older individuals, and other non-clinical populations indicate that concurrent or combined aerobic–resistance programs can improve pulmonary function, respiratory muscle strength, and functional capacity, with outcomes depending on dose, population, and protocol characteristics [23,29,34,35,65,66,67]. In addition, work in athletic populations suggests that integrating IMT with strength or technical training can enhance respiratory muscle performance, maximal strength, or sport-specific capacity [11,12,13,29,36]. Evidence from clinical and special populations is therefore used here only to support the plausibility of multi-target adaptations when RMT is integrated with peripheral resistance or aerobic training, while recognizing that the present trial specifically addresses healthy amateur runners. In this context, our data indicate that adding peripheral resistance or aerobic exercise to an RMT base was associated with short-term within-group changes across multiple respiratory and cardiopulmonary domains in amateur runners; however, these observations should be interpreted cautiously and should not be taken as definitive evidence of performance improvement or modality superiority. At the same time, because the present analyses cannot cleanly isolate treatment effects between arms, our conclusions are intentionally confined to the observation that different RMT-based combinations were associated with different patterns of within-group change, rather than asserting regimen-specific superiority.

This study has several strengths, including a randomized four-arm design, a standardized RMT protocol common to all groups, and comprehensive pre–post assessment of lung function, respiratory muscle strength, and CPET-derived outcomes in a homogeneous sample of amateur runners. There are, however, important limitations that must be emphasized. Importantly, the present analyses do not provide a definitive baseline-adjusted between-group estimate of treatment effect; therefore, the observed within-group improvements should not be interpreted as evidence that any training modality is superior to another. First, the statistical framework used in this trial—paired *t*-tests for within-group comparisons and one-way ANOVA on change scores for between-group comparisons—is not the optimal approach for a four-arm randomized pre–post design, particularly in the presence of baseline differences. Baseline-adjusted methods such as ANCOVA or linear mixed-effects models would provide a more rigorous estimation of group-by-time effects and better handling of baseline imbalances and within-subject correlation. This concern is particularly relevant for VO_2_peak, where the RMT + LLRT group started with a higher baseline value than the other arms; without ANCOVA or mixed-effects adjustment, the internal validity of the VO_2_peak between-group comparisons is therefore limited. Due to time and resource constraints, a full re-analysis using these advanced methods was not undertaken, and our findings must therefore be interpreted within the limits of the original analytic plan. Consequently, this study should be considered exploratory (pilot-scale) and hypothesis-generating; thus, all between-group comparisons should be interpreted cautiously, and any apparent differences in patterns of change between interventions cannot be considered definitive evidence of modality-specific superiority. Second, the sample size was modest (*n* = 12 per group), which reduces statistical power to detect small-to-moderate between-group differences and increases the risk of both type I and type II errors. In addition, multiple outcomes and comparisons were examined without multiplicity adjustment, which may further increase the probability of false-positive findings; thus, statistically significant within-group changes should be interpreted cautiously. Given that the a priori power analysis was based on an assumed effect size of f = 0.50 (moderate-to-large), the present trial was likely underpowered to detect smaller between-group differences, particularly among the three combined training regimens. The six-week intervention period, while sufficient to elicit short-term training adaptations, does not inform long-term maintenance of effects or potential seasonal interactions with runners’ broader training cycles. Third, although the supervised sessions were standardized, we did not quantify or control participants’ external training during the intervention (including overall running volume/intensity and any non-study strength training), which may have introduced unmeasured variability in training dose and contributed to the observed pre–post changes. In addition, although adherence was monitored using attendance logs and home record sheets, we did not calculate or report adherence as a quantitative percentage for each group, which limits precision in describing the delivered training dose. Fourth, the sample consisted of non-elite, relatively healthy amateur runners recruited from a single center, which limits the generalizability of the findings to other athletic or clinical populations, such as highly trained distance runners or individuals with cardiopulmonary disease. Finally, we did not include running performance outcomes (e.g., time-trial performance, running economy, or injury incidence) or mechanistic measures such as diaphragm imaging or chest-wall kinematics, which constrains our ability to translate the observed physiological changes into clear performance or clinical recommendations.

Future studies should address these limitations by using a priori–planned statistical models that are appropriate for multi-arm pre–post designs, such as baseline-adjusted ANCOVA or linear mixed-effects models with random intercepts for participants and fixed effects for group, time, and their interaction. Larger, adequately powered multicenter trials with longer follow-up periods are needed to determine whether the different RMT-based combinations truly differ in their long-term effects on lung function, respiratory muscle strength, and cardiopulmonary performance. Including a non-training or sham-RMT control group, stratifying randomization by key factors such as sex and baseline VO_2_peak, and pre-registering primary and secondary outcomes would further strengthen causal inference. In addition, future research should incorporate running-specific endpoints (e.g., 3–5 km time trials, running economy, race performance, fatigue and dyspnea ratings, and running-related injury incidence) alongside physiological measures, to clarify how changes in respiratory and cardiopulmonary function translate into meaningful benefits for runners. Such trials will be essential to move beyond the exploratory, hypothesis-generating nature of the present study and to establish robust, evidence-based recommendations regarding the optimal integration of RMT, resistance training, and aerobic exercise in the training programs of amateur and competitive runners.

## 5. Conclusions

In this randomized four-arm trial of 48 amateur runners, six weeks of respiratory muscle training (RMT)–based programs—implemented either alone or in combination with upper-limb resistance training (RMT + ULRT), lower-limb resistance training (RMT + LLRT), or aerobic exercise training (RMT + AET) were associated with within-group pre–post improvements in at least one of the targeted domains: lung function (forced vital capacity [FVC], forced expiratory volume in one second [FEV_1_], FEV_1_/FVC ratio), respiratory muscle strength (maximal inspiratory and expiratory pressures, MIP and MEP), or whole-body exercise capacity (peak oxygen uptake [VO_2_peak] and the VE/VCO_2_ slope). However, between-group comparisons of change scores (Δ) were not statistically significant for any outcome, and VO_2_peak differed between groups at baseline. Thus, within the constraints of the present analytic approach, the data do not demonstrate clear superiority of any single RMT-based combination over the others.

Within these limitations, our findings suggest that all RMT-based programs tested here were feasible and were associated with modest, short-term physiological adaptations in amateur runners. Because running-specific performance outcomes were not assessed and between-group Δ comparisons were not significant, any apparent domain-specific patterns should be interpreted as descriptive and exploratory rather than as evidence of performance improvement or superiority of any modality. Because of the modest sample size, short intervention duration, baseline VO_2_peak imbalances, and the use of paired *t*-tests and change-score ANOVA rather than baseline-adjusted models, these results should be interpreted as exploratory and hypothesis-generating rather than confirmatory. Future studies with larger samples, longer follow-up, more robust statistical methods (such as baseline-adjusted ANCOVA or linear mixed-effects models), and running-specific performance outcomes are needed to determine whether particular RMT-based combinations truly differ in their effects on lung function, respiratory muscle strength, and cardiopulmonary performance.

## Figures and Tables

**Table 1:** Independent and dependent variables of the study.

Independent Variable	Dependent Variable
Respiratory Muscle Training RMT + Upper-Limb Resistance Training RMT + Lower-Limb Resistance Training RMT + Aerobic Exercise Training	Respiratory function (FVC, FEV_1_, FEV_1_/FVC) Respiratory strength (MIP, MEP) Cardiopulmonary Exercise Capacity (VO_2_peak, VE/VCO_2_ Slope)

**Table 2:** General characteristics of subjects (N = 48).

Characteristics	Mean ± SD
Male/Female (%)	31/17 (64.6/35.4)
Age (year)	32.98 ± 4.00
Height (cm)	170.44 ± 9.32
Weight (kg)	69.53 ± 13.00
BMI (kg/m^2^)	23.77 ± 2.87
Sessions/week (sess/wk)	3.35 ± 1.18
Km/week (km/wk)	28.15 ± 18.33
Smoking status, *n* (%): Current	12 (25)
BMI, body mass index	

**Table 3:** Comparison of FVC among the groups (N = 48).

		RMT (*n* = 12)	RMT + ULRT (*n* = 12)	RMT + LLRT (*n* = 12)	RMT + AET (*n* = 12)	*p*
FVC (L)	Pre-test	3.87 ± 0.72	4.19 ± 1.04	4.1 ± 0.90	4.47 ± 1.02	0.48
	Post-test	4.08 ± 0.80	4.5 ± 1.11	4.4 ± 0.84	4.52 ± 0.94	0.63
	Change	0.21 ± 0.47	0.31 ± 0.28	0.3 ± 0.34	0.05 ± 0.31	0.29
	*p*	0.15	0.00 **	0.01 *	0.57	

RMT: Respiratory Muscle Training group, RMT + ULRT: RMT + Upper-Limb Resistance Training group, RMT + LLRT: RMT + Lower-Limb Resistance Training group, RMT + AET: RMT + Aerobic Exercise Training group, FVC: Forced vital Capacity, * *p* < 0.05, ** *p* < 0.01.

**Table 4:** Comparison of FEV_1_ among the groups (N = 48).

		RMT (*n* = 12)	RMT + ULRT (*n* = 12)	RMT + LLRT (*n* = 12)	RMT + AET (*n* = 12)	*p*
FEV_1_ (L)	Pre-test	3.15 ± 0.61	3.4 ± 0.70	3.46 ± 0.80	3.46 ± 0.85	0.70
	Post-test	3.24 ± 0.72	3.5 ± 0.67	3.65 ± 0.83	3.57 ± 0.87	0.58
	Change	0.08 ± 0.30	0.07 ± 0.14	0.19 ± 0.14	0.11 ± 0.24	0.51
	*p*	0.36	0.10	0.00 **	0.13	

RMT: Respiratory Muscle Training group, RMT + ULRT: RMT + Upper-Limb Resistance Training group, RMT + LLRT: RMT + Lower-Limb Resistance Training group, RMT + AET: RMT + Aerobic Exercise Training group, FEV_1_: Forced Expiratory Volume at one second, ** *p* < 0.01.

**Table 5:** Comparison of FEV_1_/FVC among the groups (N = 48).

		RMT (*n* = 12)	RMT + ULRT (*n* = 12)	RMT + LLRT (*n* = 12)	RMT + AET (*n* = 12)	*p*
FEV_1_/FVC (%)	Pre-test	81.33 ± 7.75	81.92 ± 9.06	83.67 ± 5.03	77.25 ± 8.29	0.23
	Post-test	83.50 ± 6.59	83.50 ± 8.15	84.92 ± 5.21	79.67 ± 7.08	0.29
	Change	2.17 ± 2.21	1.58 ± 1.68	1.25 ± 1.42	2.42 ± 4.78	0.74
	*p*	0.01 **	0.01 **	0.01 **	0.11	

RMT: Respiratory Muscle Training group, RMT + ULRT: RMT + Upper-Limb Resistance Training group, RMT + LLRT: RMT + Lower-Limb Resistance Training group, RMT + AET: RMT + Aerobic Exercise Training group, FEV_1_/FVC: The ratio of forced expiratory volume for 1 s to forced vital capacity, ** *p* < 0.01.

**Table 6:** Comparison of MIP among the groups (N = 48).

		RMT (*n* = 12)	RMT + ULRT (*n* = 12)	RMT + LLRT (*n* = 12)	RMT + AET (*n* = 12)	*p*
MIP (mmH_2_O)	Pre-test	71.08 ± 26.70	75.17 ± 31.43	93.92 ± 23.94	93.50 ± 32.52	0.12
	Post-test	90.00 ± 25.16	91.58 ± 27.03	105.67 ± 17.57	96.42 ± 27.28	0.41
	Change	18.92 ± 11.52	16.42 ± 22.01	11.75 ± 10.01	2.92 ± 14.37	0.07
	*p*	0.00 **	0.03 *	0.00 **	0.50	

RMT: Respiratory Muscle Training group, RMT + ULRT: RMT + Upper-Limb Resistance Training group, RMT + LLRT: RMT + Lower-Limb Resistance Training group, RMT + AET: RMT + Aerobic Exercise Training group, MIP: Maximal inspiratory pressure, * *p* < 0.05, ** *p* < 0.01.

**Table 7:** Comparison of MEP among the groups (N = 48).

		RMT (*n* = 12)	RMT + ULRT (*n* = 12)	RMT + LLRT (*n* = 12)	RMT + AET (*n* = 12)	*p*
MEP (mmH_2_O)	Pre-test	71.08 ± 26.70	66.33 ± 20.74	83.75 ± 13.84	77.50 ± 19.76	0.20
	Post-test	91.17 ± 24.25	94.08 ± 24.07	105.67 ± 17.57	96.42 ± 27.28	0.47
	Change	10.92 ± 17.97	16.92 ± 14.21	15.08 ± 15.32	2.17 ± 20.16	0.17
	*p*	0.00 **	0.00 **	0.00 **	0.00 **	

RMT: Respiratory Muscle Training group, RMT + ULRT: RMT + Upper-Limb Resistance Training group, RMT + LLRT: RMT + Lower-Limb Resistance Training group, RMT + AET: RMT + Aerobic Exercise Training group, MEP: Maximal expiratory pressure, ** *p* < 0.01.

**Table 8:** Comparison of VO_2_peak among the groups (N = 48).

		RMT (*n* = 12)	RMT + ULRT (*n* = 12)	RMT + LLRT (*n* = 12)	RMT + AET (*n* = 12)	*p*
VO_2_peak	Pre-test	36.17 ± 6.58	37.25 ± 6.84	43.48 ± 6.06	40.26 ± 6.08	0.03 *
(mL·kg^−1^·min^−1^)	Post-test	40.76 ± 6.38	40.86 ± 6.27	48.58 ± 7.29	46.04 ± 5.99	0.01 **
	Change	4.59 ± 1.57	3.61 ± 1.53	5.10 ± 5.5	5.78 ± 3.49	0.47
	*p*	0.00 **	0.00 **	0.01 **	0.00 **	

RMT: Respiratory Muscle Training group, RMT + ULRT: RMT + Upper-Limb Resistance Training group, RMT + LLRT: RMT + Lower-Limb Resistance Training group, RMT + AET: RMT + Aerobic Exercise Training group, VO_2_peak: peak oxygen uptake, * *p* < 0.05, ** *p* < 0.01.

**Table 9:** Comparison of VE/VCO_2_ Slope among the groups (N = 48).

		RMT (*n* = 12)	RMT + ULRT (*n* = 12)	RMT + LLRT (*n* = 12)	RMT + AET (*n* = 12)	*p*
VE/VCO_2_ Slope	Pre-test	24.75 ± 2.29	25.84 ± 3.36	25.52 ± 3.75	24.68 ± 2.53	0.74
	Post-test	24.28 ± 2.84	23.90 ± 2.85	22.83 ± 1.99	23.27 ± 2.04	0.49
	Change	−0.48 ± 3.45	−1.94 ± 1.27	−2.68 ± 2.42	−1.42 ± 1.49	0.14
	*p*	0.64	0.00 **	0.00 **	0.01 **	

RMT: Respiratory Muscle Training group, RMT + ULRT: RMT + Upper-Limb Resistance Training group, RMT + LLRT: RMT + Lower-Limb Resistance Training group, RMT + AET: RMT + Aerobic Exercise Training group, VE/VCO_2_ Slope: ventilatory efficiency, ** *p* < 0.01.

## Data Availability

The data presented in this study are available on request from the corresponding author due to privacy and ethical restrictions related to participant confidentiality and the terms of IRB approval.
